# Causes of the Racial Disparities in the Risk of Alzheimer’s Disease

**DOI:** 10.1093/geroni/igab046.227

**Published:** 2021-12-17

**Authors:** Stanislav Kolpakov Nikitin, Arseniy Yashkin, Julia Kravchenko, Igor Akushevich

**Affiliations:** 1 Duke University, Durham, North Carolina, United States; 2 Duke University, Morrisville, North Carolina, United States

## Abstract

The risk of Alzheimer’s disease (AD) is not uniform across race-specific subpopulations: Blacks face approximately 50% higher risk of AD onset compared to Whites(Hazard Ratio=1.50; 95%CI:1.46-1.54). We used Blinder-Oaxaca decomposition, modified for censored data, to explain the disparities in the risk of AD between these races in Medicare beneficiaries aged 65+. This approach measures the contributions to the total difference in AD risks from the differences in the prevalence and the difference in magnitude of the effects of each potential explanatory variable. We used hypertension, diabetes mellitus, depression, cerebrovascular and renal diseases as the potential causes of the racial disparities in AD risk. We found that the greatest contribution was due to the impact of arterial hypertension, of which 24% of the effect was due to differences in prevalence and 76% due to the differences in effect magnitude. Unexpectedly, the contributions of other studied diseases explained only a small part of the racial disparity in AD risk. The remaining incidence rates, which could not be explained by the contributions of hypertension and other included diseases in the age-specific analysis, were lower for the Black population, although initially, the total age-specific incidence rates of AD were higher for the Blacks when compared to the Whites. Therefore, our results suggest that targeted interventions in the Black subpopulation are urgently needed to mitigate the adverse health effects of hypertension, independent of the possible causes, such as access to hypertension care, or race-related differences in adherence to antihypertensive treatment.

